# Field evaluation (FE) of the World Health Organization (WHO) interim guidelines (IG) on infection prevention and control (IPC) of epidemic and pandemic-prone acute respiratory diseases (ARD) in health care

**DOI:** 10.1186/1753-6561-5-S6-P280

**Published:** 2011-06-29

**Authors:** J Conly, R Thakur, S Eremin, C Pessoa Silva

**Affiliations:** 1 Global Alert and Response, Infection Prevention and Control in Health Care, WHO, Geneva, Switzerland

## Introduction / objectives

A FE was designed with the objective of assessing the feasibility and applicability of the IG and the use of implementation tools in healthcare facilities (HCFs) over an initial period of 6-9 months and to apply lessons learnt to improve further versions of the IG.

## Methods

Definitions for feasibility and applicability were based on the AGREE instrument. The FE design was a parallel, two-arm before-after descriptive study in 2 convenience samples, Principal sites, invited by the 6 WHO Regional Offices and voluntary Complementary sites of selected HCFs, conducted over 4 phases. A suite of tools to assist implementation, assessment, training, education and feedback were provided. Returned feedback forms were entered into Excel (Microsoft Corporation 2003) and descriptive epidemiologic analyses were conducted.

## Results

A total of 13 sites from 7 countries (4 WHO Regions) participated. The results suggested that the sites were well prepared with respect to having IPC protocols for ARDs (100%) and 92.3% sites had policies or IPC guidelines for HCF epidemic /pandemic planning available at the local and/or national level. For the surveyed HCFs, the follow-up FE revealed an overall increase (p= 0.01) to 86.8% vs the pre-survey results of 71.9%, with 10/ 15 recommendations implemented 2^o^ to the IG (p=NS). For the surveyed ICPs, the follow-up FE revealed an overall increase (p< 0.0001) to 85.9% of 72.2%, with 12/32 implemented 2^o^ to the IG (p=NS).

## Conclusion

Although the implemented changes were not significantly attributed to be as a direct result of the IG, there was an overall significant uptake of the IG recommendations and it remains possible that the changes noted were indirectly influenced by IG.

## Disclosure of interest

None declared.

